# Early Treatment with Empagliflozin and GABA Improves *β*-Cell Mass and Glucose Tolerance in Streptozotocin-Treated Mice

**DOI:** 10.1155/2019/2813489

**Published:** 2019-07-30

**Authors:** Caroline Daems, Sophie Welsch, Hasnae Boughaleb, Juliette Vanderroost, Annie Robert, Etienne Sokal, Philippe A. Lysy

**Affiliations:** ^1^Pôle PEDI, Institut de Recherche Expérimentale et Clinique, UCLouvain, Av. Hippocrate 10, B-1200 Brussels, Belgium; ^2^Pôle d'Epidémiologie et Biostatistique, Institut de Recherche Expérimentale et Clinique, UCLouvain, Av. Hippocrate 10, B-1200 Brussels, Belgium

## Abstract

While the autoimmune character of T1D (type 1 diabetes) is being challenged, it is currently recognized that inflammation plays a key role in its development. We hypothesized that glucotoxicity could contribute to *β*-cell mass destruction through participation in islet inflammation. We evaluated the potential of empagliflozin (EMPA) and GABA (gamma-aminobutyric acid) to protect *β*-cell mass against glucotoxicity and to increase *β*-cell mass after diagnosis of T1D. Empagliflozin is a SGLT2 (sodium-dependent glucose cotransporter) inhibitor which thereby blocks glucose recapture by the kidney and promotes glucose excretion in urine. GABA is an inhibitory neurotransmitter, which stimulates *α*-to-*β* cell transdifferentiation. In streptozotocin-treated mice, empagliflozin and/or GABA were delivered for a period of five days or three weeks. As compared to untreated T1D mice, EMPA-treated T1D mice had decreased FFA (free fatty acid) levels and improved glucose homeostasis. EMPA-treated T1D mice had higher islet density, with preserved architecture, compared to T1D mice, and EMPA-treated T1D mice also differed from T1D mice by the total absence of immune cell infiltration within islets. Islets from EMPA-treated mice were also less subjected to ER (endoplasmic reticulum) stress and inflammation, as shown by qPCR analysis. Glucose homeostasis parameters and islet area/pancreas area ratio improved, as compared to diabetic controls, when T1D mice were treated for three weeks with GABA and EMPA. T1D EMPA+GABA mice had higher glucagon levels than T1D mice, without modifications of glucagon area/islet area ratios. In conclusion, empagliflozin and GABA, used in monotherapy in streptozotocin-induced diabetic mice, have positive effects on *β*-cell mass preservation or proliferation through an indirect effect on islet cell inflammation and ER stress. Further research is mandatory to evaluate whether empagliflozin and GABA may be a potential therapeutic target for the protection of *β*-cell mass after new-onset T1D.

## 1. Introduction

Type 1 diabetes (T1D) results from the progressive destruction of pancreatic *β* cells by the immune system related to inflammation called insulitis, and it clinically develops when *β*-cell mass drops to 10-15% of its initial value [1]. Administration of insulin leads to a recovery of *β*-cell function in about 60% of patients [2], and the resurgence of endogenous insulin secretion (determined via C-peptide levels) leads to a decrease in daily insulin requirements and to lower blood glucose values with better disease control. This so-called “honeymoon period,” which is a preamble to the future demise of *β* cells, offers a unique possibility for intervention trials aiming at the preservation of *β*-cell mass. Yet this remains an unmet need since current strategies for *β*-cell mass preservation have globally failed to show significant effects on disease control (*i.e.*, HbA_1C_ levels), especially when focusing on immunotherapy [3, 4]. On the other hand, several drugs, evaluated mostly in models of type 2 diabetes (T2D), were encouraging in their potential to improve *β*-cell mass and function either through a mechanism of reduced metabolic toxicity (*e.g.*, SGLT2 inhibitors) or through the replenishment of the *β*-cell compartment (*e.g.*, GABA). Some of these drugs are particularly appealing because of their availability in medicine, and their antidiabetic properties should be evaluated in single or combinatory protocols.

For several years, gliflozins such as empagliflozin, a SGLT2 inhibitor, appear as a useful approach to alleviate hyperglycemia in patients with diabetes. SGLT2 are low-affinity, high-capacity glucose transporters located in the renal proximal tubule. These transporters are involved in 90% of the glucose reabsorption in the kidney and therefore represent an important therapeutic target for treating diabetes. Several studies already suggested the potential of SGLT2 inhibitors (gliflozins) to improve glucose control in patients with T2D [5] and insulin-treated patients with T1D [6–8]. Besides this emerging clinical evidence, little is known about the impact of SGLT2 inhibitors (gliflozins) on pancreatic islet inflammation. Improvement of insulin sensitivity was induced in *db*/*db* mice after treatment with empagliflozin, which was correlated to lower levels of inflammatory response, fatty acid synthesis, and oxidation in these animals [9]. Recently, empagliflozin, a member of the gliflozin family, was shown to increase *β*-cell mass and proliferation in mice submitted to *β*-cell ablation with streptozotocin [10]. Furthermore, two pilot studies from the same group evaluated the effects of empagliflozin in patients with impaired fasting glucose [11] or T2D [12] and showed an improvement of *β*-cell function during hyperglycemic clamps only after two weeks of treatment. Together, these studies provide arguments for a beneficial impact on *β*-cell mass and/or function of a specific glucose-lowering therapy and fuel the need to evaluate the *β*-cell mass protection effects of SGLT2 inhibitors during the development of T1D.

A 2017 study by Ben-Othman et al. [13] revealed the unexpected potential of GABA to stimulate *β*-cell regeneration through the recruitment of cells inside the *α*-cell pool that undergo transdifferentiation after the repression of *Arx* expression, in a context of *β*-cell ablation. These findings provoked a deep interest in the GABA molecule, particularly because the processes of *β*-cell regeneration are independent of disease mechanisms and could be applied both to T1D and T2D. There is currently a blossoming of preclinical and clinical studies (NCT02002130, NCT01917760), some of which are still actively recruiting patients with T1D (NCT03635437, NCT01781884). Although controversy recently arose regarding the reproducibility of the preclinical protocols [14] and whereas it is actually unclear as to whether patients chronically under GABA receptor agonists may be protected to some extent against diabetes, a recent clinical study [15] suggested that GABA may reduce the proinflammatory profiles of peripheral blood mononuclear cells and CD4^+^ T cells from patients with T1D. This immunomodulatory aspect, if confirmed *in vivo*, may be an added value for the maintenance of newly formed *β*-cells in unfavorable immune or inflammatory backgrounds.

The goal of our study was to investigate the potential of empagliflozin, a SGLT2 inhibitor, for prolonging *β*-cell survival and function in a mouse model of T1D. The adjunctive effects of GABA were also evaluated in combination protocols for potential in alleviating the metabolic stress of *β* cells and fostering their survival immediately after T1D onset.

## 2. Materials and Methods

### 2.1. Animals

Six- to eight-week-old male NMRI mice (30-40 g) were purchased from Charles River Laboratories Deutschland Inc. (Sulzfeld, Deutschland). Mice were fed with standard laboratory diet and water *ad libitum*. The study was approved by the UCLouvain Ethical Committee (Brussels, Belgium; permit number: 2015/UCL/MD/07) and was conducted in accordance with the Practice Guidelines for Laboratory Animals of Belgium. NMRI mice are a well-known mice model for streptozotocin-induced T1D [16–18]. Moreover, the multiple low-dose STZ-induced diabetes mice model is a model widely used to demonstrate the effect of drug treatment or knockdown in type 1 diabetic mice. There were various publications about this model in T1D treatment research development such as the works of Koya et al. [19], Zhang et al. [20], Cheng et al. [10], and Pighin et al. [21].

### 2.2. *In Vivo* Protocols

T1D was induced by the intraperitoneal injection of streptozotocin at 50 mg/kg body weight (BW) for four consecutive days [22, 23]. Control mice were injected with the vehicle of streptozotocin (sodium citrate buffer 0.1 M, pH 4.4). After the injection, the mice rested for three days for the development of overt diabetes (considered as blood glucose >250 mg/dL). Two studies were conducted to test the molecules of interest in the treatment of T1D: protocol #1 evaluated the effects of empagliflozin during a five-day exposure (short duration, sd) and protocol #2 evaluated the effects of GABA and empagliflozin during a three-week-duration treatment (long duration, ld). Protocol #1 consisted of three groups of mice: (1) control mice (CTL), (2) diabetic mice treated with vehicle (Natrosol 0.5%) (T1D), and (3) diabetic mice treated with 10 mg/kg BW empagliflozin (T1D EMPA). Protocol #2 consisted of five groups of mice: (1) control mice (CTL), (2) diabetic mice treated with vehicle (NaCl 0.9%) (T1D), (3) diabetic mice treated with 10 mg/kg BW empagliflozin (T1D EMPA), (4) diabetic mice treated with 10 mg/kg BW GABA (T1D GABA), or (5) diabetic mice treated with 10 mg/kg BW empagliflozin and GABA (T1D EMPA+GABA).

The treatment with empagliflozin was delivered daily to the mice by oral gavage and GABA was administered daily by intraperitoneal injection. Capillary blood glucose (FreeStyle Precision Neo, Abbott) and body weight were measured every day. When blood glucose leveled at above 400 mg/dL, diabetic mice were subcutaneously injected with 6 IU of human glargine insulin (Lantus®, Lilly). All mice were sacrificed at the end of the protocols by cervical dislocation after anesthetic injection.

### 2.3. Intraperitoneal Glucose Tolerance Test (IPGTT)

IPGTTs were performed at the end of the treatment protocols (day 12 or 28). After a 16 h fast, 2 mg/g BW of a solution of 20% D-glucose (Sterop) was injected intraperitoneally to the mice. Blood was drawn from the tail vein, and blood glucose levels were measured at 0, 15, 30, 60, and 120 min after the injection using a glucometer (FreeStyle Precision Neo, Abbott).

### 2.4. Multiplex

The multiplex assay used was a multiple ELISA assay. Indeed, thanks to magnetic beads coated for different antibodies, it is possible to perform an ELISA for several antigens for the same sample in the same well. The multiplex assay was performed using a Milliplex kit (MMHMAG-44K, Millipore, Merck) following the manufacturer's instructions. Briefly, samples were incubated with a magnetic bead mix (directed against glucagon and TNF*α*) overnight at 4°C under agitation. The plate was washed three times with 1x wash buffer, and 50 *μ*L of a solution containing detection antibodies was added to each well. The plate was incubated for 30 min under agitation at room temperature (RT). Fifty microliters of a streptavidin-phycoerythrin solution was added into each well, and the plate was incubated under agitation for 30 min at RT. The plate was washed three times, 100 *μ*L sheath fluid was added into each well, and the plate was read using the Bio-Rad Luminex® 2000™ software. The gate settings were set according to the manufacturer's instructions (i.e., 8,000 to 15,000).

### 2.5. Biochemical Analysis (Blood Analysis)

Blood samples were collected with heparinized syringes during sacrifice. FFA levels were measured by the medicine laboratory at KULeuven (Leuven, Belgium) using a colorimetric test (Diagnostic Systems #1 5781 99 10 935).

### 2.6. RNA Extraction and Quantitative RT-PCR

Total RNA was isolated from the mouse pancreas tissues using Lysing Matrix D tubes (MP Biomedicals #6913-100) and the TriPure reagent (Sigma-Aldrich). Briefly, a piece of pancreas was ground in the TriPure reagent using the ceramic balls contained in the Lysing Matrix D tubes and using the FastPrep grinder two times (6 m/s for 40 sec). Total RNA extraction was performed from one quarter of this homogenate following the TriPure manufacturer's instructions. First-strand cDNA was synthesized from a 1 *μ*g aliquot of the total RNA using a commercial reverse transcription kit (Applied Biosystems, Thermo Fisher Scientific). RT-qPCR was performed using SYBR Green (Thermo Fisher Scientific, #4367659), the StepOne Plus Real-Time PCR System, and StepOne Software v2.1 (Applied Biosystems). Quantitative RT-PCR was performed according to these following conditions: one denaturation step for 10 min at 95°C followed by 40 cycles of denaturation (15 sec at 95°C), annealing (30 sec at 55°C), and extension (30 sec at 60°C) for *Xbp1s*, *Atf4*, *Bip*, *Il6*, *iNos*, and *Il1β*. Annealing conditions were 15 sec at 58°C for *Txnip*. *Rpl19* was used as an internal control and its annealing conditions were 15 sec at 62°C. Primers for qPCR are listed in Table S1.

### 2.7. Histological Analysis

Pancreas tissues were fixed overnight in 4% paraformaldehyde (PFA) and embedded in paraffin. Then, the samples were sectioned into 5 *μ*m thick slices, mounted on glass slides, and colored with hematoxylin-eosin (H&E). Images were acquired using a Leica SCN400 Slide Scanner (Leica, Germany) and then analyzed with Digital Image Hub software (Leica, Germany). For immunofluorescence, sections were blocked in 10% bovine serum albumin (Sigma-Aldrich), 3% nonfat milk, and 0.3% Triton X-100 PBS. For the detection of insulin and glucagon, tissues were incubated overnight at 4°C with antibodies (listed in Table S2) diluted in PBS/0.3% Triton X-100/10% BSA/3% milk. Then, tissues were incubated the next day for 2 h at RT in the dark with the respective secondary antibodies (listed in Table S2) diluted in PBS/0.3% Triton X-100/10% BSA. After washing, tissues were incubated for 5 min with DAPI (4′,6-diamidino-2-phenylindole) diluted at the final concentration of 0.2 *μ*g/mL in distilled water. Then, sections were mounted with Faramount Mounting Medium (Dako, # S3025) and observed under a fluorescence microscope (40x/0.75; DAPI: 50 ms; insulin: 250 ms; and glucagon: 350 ms). The digital images were acquired by a digital imaging platform (3DHistech Pannoramic P250 Flash III, nicknamed Oyster) and analyzed using the Visiopharm software (Visiopharm A/S). For regular/irregular islet identification, the following was used:

(1) $$\begin{array}{c} \text{Convexity}=\frac{{\text{perimeter}}_{\text{c},\text{s}}}{{\text{perimeter}}_{\text{s}}}, \end{array}$$

where perimeter_c,s_ is the perimeter of the simplified object of the convex hull of an object and perimeter_s_ is the perimeter of the simplified object. The simplified object was a continuous representation of objects instead of the pixelated object and was created using polygon simplification, which reduces the influence of pixilation. Islets were considered irregular when the convexity was ≤0.72.

### 2.8. Statistical Analysis

Statistical analyses were performed as follows: first, we confirmed whether the populations were normally distributed using the Shapiro-Wilk test. If the population was distributed normally, we did an ANOVA-Tukey test. However, if the assumption of equality of variance (Bartlett's test) is rejected, we performed an ANOVA-Welch test. If the population was not distributed normally, we performed a Kruskal-Wallis test. For the qPCR, the significance between the T1D group and the T1D EMPA group was assayed using a *t*-test. Data were expressed as mean ± standard error of the mean. *p* < 0.05 was considered significant for all statistical analyses. The statistical analyses were performed using the SPSS Statistics or the GraphPad Prism (GraphPad Software Inc.) software. GraphPad Prism (GraphPad Software Inc.) was also used to perform a Kaplan-Meyer curve and its statistics (log-rank test). For this analysis, mice were considered diabetic when blood glucose exceeded 200 mg/dL for two consecutive days [24].

## 3. Results

In our current investigations for the protection of *β* cells against glucotoxicity, we used STZ-treated mice as a type 1-like insulin-deficient diabetes model. Diabetic animals were treated with (1) the SGLT2 inhibitor empagliflozin, (2) the neurotransmitter GABA, or (3) a combination of the two drugs.

**Figure 1:**
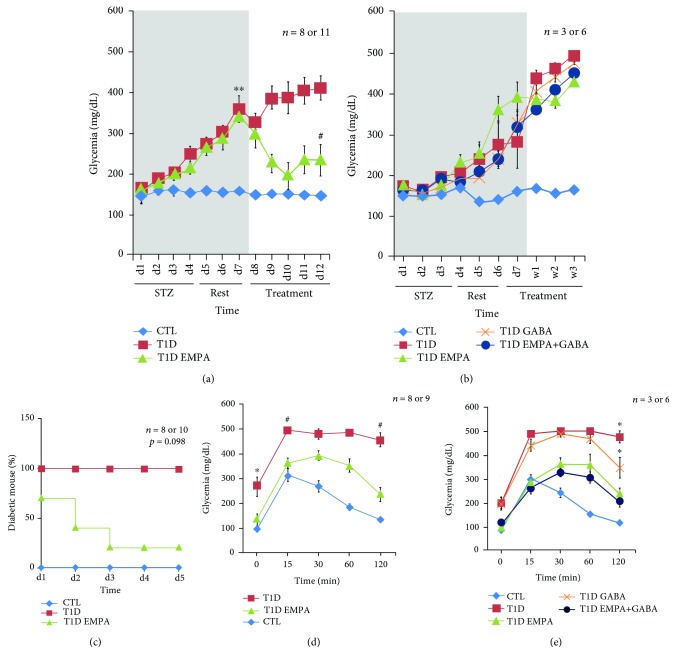
Glycemia evolution and the effect of empagliflozin, GABA, and their combination on the homeostasis of glucose. Empagliflozin treatment decreases glucose level and diabetic mice percentage. Long-duration treatments of empagliflozin and GABA improve glucose tolerance of diabetic mice. (a and b) The grey area corresponds to the STZ injection period (four days) and the rest period (three days). (a) Asterisks (^∗∗^) show a significant difference between the CTL group and the T1D group (*p* = 0.002, ANOVA-Welch) and between the CTL group and the T1D EMPA group (*p* = 0.004, ANOVA-Welch). Hashtag (#) shows a significant difference between the T1D group and the T1D EMPA group (*p* = 0.032, Kruskal-Wallis). (b) Statistics performed using a Kruskal-Wallis test. (a) and (b) correspond to protocols #1 and #2, respectively. The letters d and w correspond to day and week, respectively. (c) For protocol #1, the Kaplan-Meyer curves represent the percentage of diabetic mice from the first day of treatment to the day of sacrifice. Statistics performed using a log-rank test. Mice were considered diabetic when blood glucose exceeded 200 mg/dL for two consecutive days. (d and e) An intraperitoneal glucose tolerance test (IPGTT) was performed at the end of the treatments. Blood glucose was measured at different times after a 20% glucose injection in mice. (d) Protocol #1: asterisk (^∗^) shows a significant difference (*p* < 0.011) between the CTL group and the T1D group. Hashtag (#) shows a significant difference (*p* < 0.001 and *p* < 0.05, respectively) between the CTL and T1D EMPA groups and the T1D group. (e) Protocol #2: asterisk (^∗^) shows a significant difference (*p* ≤ 0.05) between the CTL, T1D EMPA, and T1D EMPA+GABA groups and the T1D and T1D GABA groups. (d and e) Statistical analyses were performed using a Kruskal-Wallis test. Glycemia was monitored for control mice (CTL, diamond blue curve); untreated diabetic mice (T1D, square red curve); and diabetic mice treated with empagliflozin (T1D EMPA, triangle green curve), GABA (T1D GABA, cross orange curve), or empagliflozin and GABA (T1D EMPA+GABA, round marine blue curve). The bars represent the standard error of the mean. For each protocol, the number of mice per group is indicated (*n* = 8 or 11, *n* = 8 or 10, *n* = 8 or 9, and *n* = 3 or 6).

Throughout the experiments, blood glucose monitoring served as a screening for diabetes emergence, evolution, and treatment efficacy (Figure 1). In protocol #1, diabetes appeared at day 3 (d3) (>250 mg/dL) and glycemia increased until drug administration at d8 in the T1D group and the T1D EMPA group (*p* = 0.002 and *p* = 0.004, respectively) compared to the control mice. Diabetic mice treated with empagliflozin alone (short duration; *i.e.*, T1D EMPA_sd_) had decreased glycemia from d12 compared to T1D mice (*p* = 0.032) (Figure 1(a)). However, at the end of treatment (w3), EMPA and GABA (long-duration treatment; *i.e.*, T1D EMPA_ld_ and T1D GABA_ld_) did not improve glucose levels of diabetic mice (*p* = 0.161 and *p* = 1.000, respectively) compared to untreated diabetic mice (Figure 1(b)). Furthermore, 71% of mice in the T1D EMPA_sd_ group reverted to normoglycemia. The percentage decrease of diabetic mice was significant (*p* = 0.036), whereas the difference between the curves were not significant (*p* = 0.098) (Figure 1(c)). Diabetic mice treated for a long duration did not revert to normoglycemia (data not shown). These results suggested that empagliflozin influences glucose levels through the induction of glucosuria and/or through *β*-cell mass protection.

To investigate *β*-cell mass functionality in diabetic mice within treatment groups, IPGTTs were performed before sacrifice. At *T* = 0, the glycemia level of the untreated diabetic mice (T1D) was higher than the glycemia level of the control mice (CTL) (*p* = 0.011). At *T* = 15, T1D mice had a higher glycemia level than CTL mice (*p* < 0.001) or T1D EMPA_sd_ mice (*p* = 0.009). At the end of the test, the glycemia level of the T1D EMPA_sd_ mice was normalized compared to those of the CTL mice (*p* = 0.335). However, the glycemia level of the T1D mice did not normalize compared to those of the CTL mice (*p* < 0.001) and the T1D EMPA mice (*p* = 0.023) (Figure 1(d)). When mice were treated with empagliflozin alone or in combination with GABA for 3 weeks (long-duration treatment; *i.e.*, T1D EMPA_ld_ and T1D EMPA+GABA_ld_), glycemia decreased to basal levels at the end of IPGTT (Figure 1(e)) compared to the CTL mice (*p* = 0.066 and *p* = 1.000), suggesting that mice within the T1D EMPA and T1D EMPA+GABA_ld_ groups had normal glucose tolerance.

To understand if glucose tolerance improvement is due to a residual insulin secretion, we assayed insulin levels in blood during IPGTT at time 0, 30, and 60 min (Figure S1). CTL mice had a very low insulin level at 0 min (0.17 ± 0.10 *μ*g/L) then the insulin level increased at 30 min (9.04 ± 4.55 *μ*g/L) and started to decrease at 60 min (6.66 ± 5.28 *μ*g/L). T1D mice had high insulin levels at 0 and 30 min (4.10 ± 2.61 and 6.92 ± 3.27 *μ*g/L, respectively) then the insulin level decreased drastically at 60 min (0.27 ± 0.07 *μ*g/L). Mice of the T1D EMPA_ld_ group had a low insulin level at 0 and 30 min (1.19 ± 0.54 and 1.27 ± 0.63 *μ*g/L, respectively) then they had a peak of insulin at 60 min (6.33 ± 2.40 *μ*g/L). T1D GABA_ld_ mice had an insulin level of approximately 3 *μ*g/L at 0 and 30 min (3.48 ± 2.69 and 3.01 ± 2.72 *μ*g/L, respectively) then the insulin level peaked at 60 min (6.05 ± 3.38 *μ*g/L). T1D EMPA+GABA_ld_ mice had constant insulin levels at 0, 30, and 60 min (1.32 ± 0.42, 1.80 ± 0.97, and 1.21 ± 0.50 *μ*g/L, respectively). The insulin level of T1D EMPA_ld_ and T1D GABA_ld_ mice had a similar profile with that of the CTL mice, whereas they had an insulin peak offset by 30 min (Figure S1 A). T1D mice had a high insulin level, inconsistent with the fact that they were diabetic, which may be explained by the fact that T1D mice (untreated or treated) had received exogenous human insulin (Figures S1 B and S1 C), which was detected by the ELISA kit used (data not shown).

**Figure 2:**
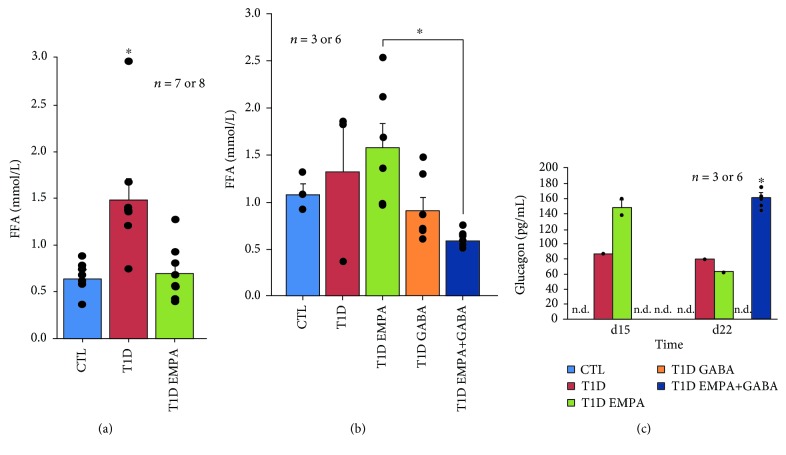
Effect of empagliflozin, GABA, and their combination on free fatty acid and glucagon levels. Blood samples of the control mice (CTL, blue bar); untreated diabetic mice (T1D, red bar); and mice treated with empagliflozin (T1D EMPA, green bar), GABA (T1D GABA, orange bar), or empagliflozin and GABA (T1D EMPA+GABA, navy blue bar) were collected at different time points: at the end for FFA dosage (a and b) and at d15 and d22 for glucagon dosage by multiplex (c). (a) Protocol #1: asterisk (^∗^) indicates a statistically significant difference (*p* = 0.009 and *p* = 0.008, respectively; Kruskal-Wallis) between the T1D group and the CTL and T1D EMPA groups. (b) Protocol #2: asterisk (^∗^) shows that there is a significant difference (*p* = 0.011; Kruskal-Wallis) between the T1D EMPA group and the T1D EMPA+GABA group. (c) n.d. means undetectable. Asterisk (^∗^) indicates a statistically significant difference (*p* < 0.05; Kruskal-Wallis) between the CTL, T1D EMPA and T1D GABA groups, and the T1D EMPA+GABA group. Each black point represents an individual. The bars represent the standard error of the mean. For each protocol, the number of mice per group is indicated (*n* = 7 or 8, *n* = 3 or 6).

When reaching a critical level, *β*-cell loss and insulinopenia lead principally to hyperglycemia; however, they can also lead to hyperlipidemia by excessive lipid degradation in free fatty acids (FFAs) [25]. The long-term toxic effects of high levels of glucose and FFAs on *β*-cell function and survival are well known [26, 27]. In our *in vivo* experiments, plasma FFA levels in T1D mice were increased compared to those of CTL (1.48 ± 0.23 vs. 0.64 ± 0.097 mmol/L; *p* = 0.009) and T1D EMPA_sd_ (0.70 ± 0.10 mmol/L; *p* = 0.008) mice (Figure 2(a)). Furthermore, FFA levels decreased in EMPA+GABA_ld_-treated mice (0.90 ± 0.15 mmol/L; *p* = 0.011) (Figure 2(b)). These results indicate that empagliflozin-treated and GABA_ld_-treated mice had a normal lipid metabolism and might thus be insulin sufficient. To confirm these hypotheses, we evaluated glucagon levels using multiplex assays. At d1 and d8, glucagon levels were too low to be detected by a multiplex kit (data not shown). The glucagon levels of T1D and T1D EMPA_ld_ mice at d15 and d22 had increased compared to those at d1 and d8. Interestingly, at d22 T1D EMPA+GABA_ld_ mice had a higher glucagon level than the other diabetic mice which had undetectable glucagon levels (*p* < 0.05) (Figure 2(c)).

**Figure 3:**
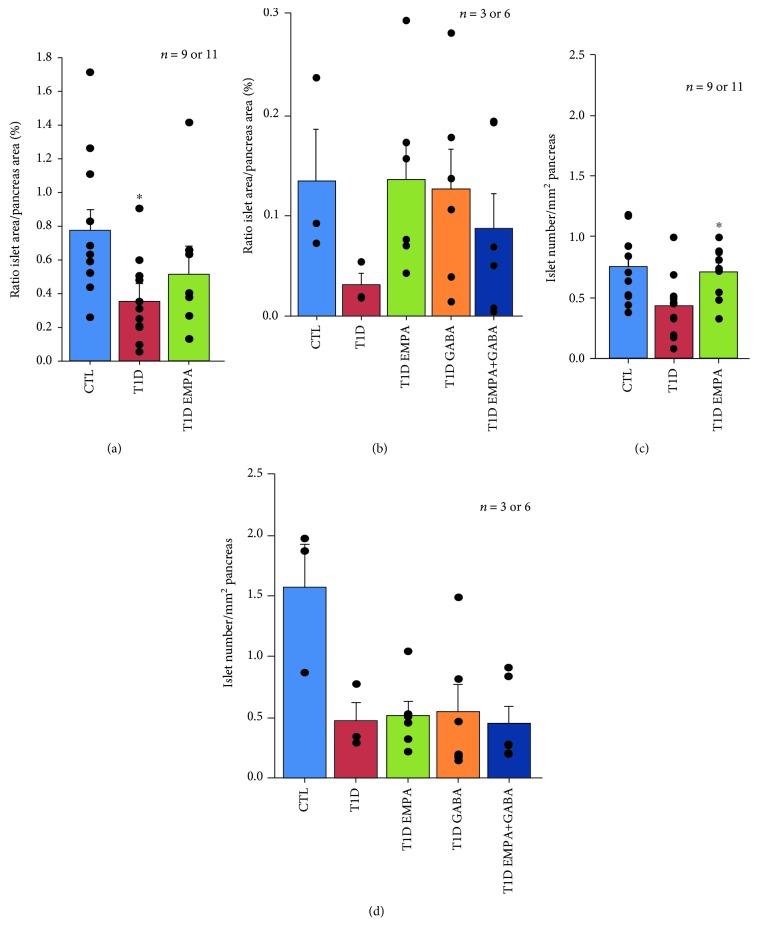
Effect of empagliflozin, GABA, and their combination on islet density and number. At the end of the treatment, the pancreas of the control mice (CTL, blue bar); untreated diabetic mice (T1D, red bar); and mice treated with empagliflozin (T1D EMPA, green bar), GABA (T1D GABA, orange bar), or empagliflozin and GABA (T1D EMPA+GABA, navy blue bar) were collected. The pancreas was divided into three parts, and its tail was used for hematoxylin and eosin coloration. The islet numbers (a and b) and the islet and pancreas areas (c and d) were calculated thanks to the Digital Image Hub software from Leica. (a) and (c) or (b) and (d) correspond to protocols #1 or #2, respectively. (a) Asterisk (^∗^) shows a significant difference (*p* = 0.038; ANOVA-Tukey) between the T1D EMPA group and the T1D group. (c) Asterisk (^∗^) shows a significant difference (*p* = 0.014; ANOVA-Tukey) between the T1D group and the T1D EMPA group. (b and d) Statistics were performed using a Kruskal-Wallis test. Each black point represents an individual. The bars represent the standard error of the mean. For each protocol, the number of mice per group is indicated (*n* = 9 or 11, *n* = 3 or 6). For each mouse, two slides with two tissue sections were analyzed.

To evaluate whether these results could be accounted on the protective effect of empagliflozin and GABA on islet cell density, mass, and proportion, we calculated the relative islet-to-pancreas proportions (islet density/mm^2^ and islet area/total pancreas area), as described elsewhere [28]. We observed that T1D EMPA_sd_ (0.51 ± 0.17%) mice had islet area/pancreas area ratios that were comparable to CTL mice (0.77 ± 0.13%) but significantly increased compared to T1D mice (0.36 ± 0.11%; *p* = 0.038) (Figure 3(a)). Furthermore, GABA_ld_, EMPA_ld_, and EMPA+GABA_ld_ treatments increased islet area/pancreas area ratios (0.13 ± 0.0004, 0.14 ± 0.0004, and 0.09 ± 0.0004%, respectively) compared to T1D mice (0.03 ± 0.001%) (Figure 3(b)). Furthermore, the islet number/pancreas area ratios were higher in mice within the T1D EMPA_sd_ (0.71 ± 0.07 islet number/mm^2^; *p* = 0.014) group compared to those in mice within the T1D (0.43 ± 0.08 islet number/mm^2^) group (Figure 3(c)). Nevertheless, EMPA_ld_ (0.51 ± 0.12 islet number/mm^2^; *p* = 0.137) or GABA_ld_ (0.55 ± 0.22 islet number/mm^2^; *p* = 0.790) treatment did not improve islet number/pancreas area ratios compared to T1D mice (0.47 ± 0.15 islet number/mm^2^) (Figure 3(d)).

**Figure 4:**
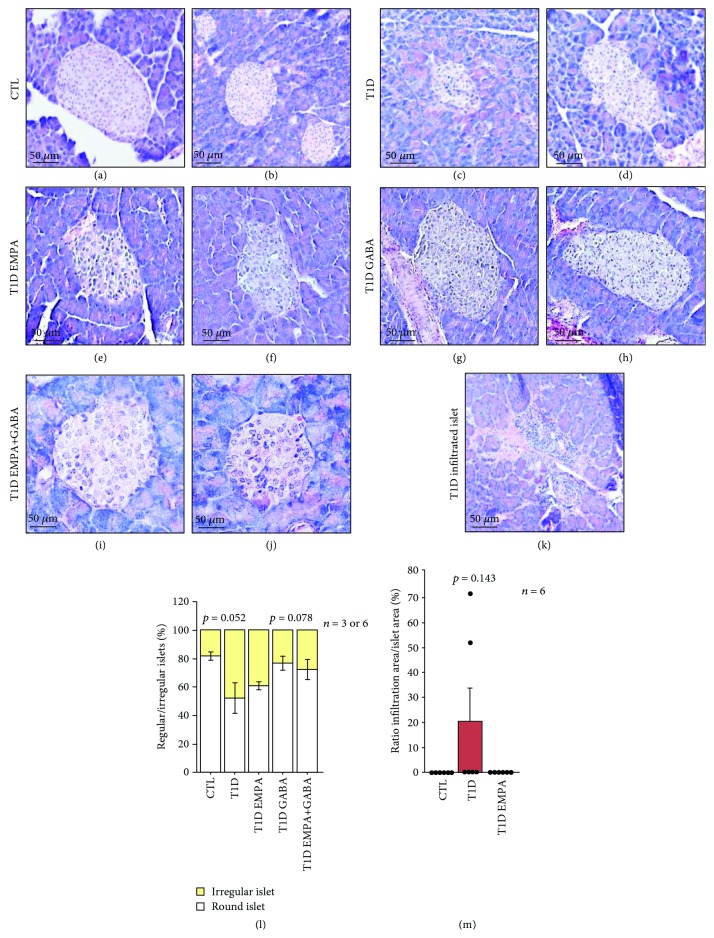
Effect of empagliflozin, GABA, and their combination on islet morphology. At the end of the treatment, the pancreas of control mice (CTL) (a and b); untreated diabetic mice (T1D) (c, d, and k); and mice treated with empagliflozin (T1D EMPA) (e and f), GABA (T1D GABA) (g and h), and both drugs (T1D EMPA+GABA) (i and j) were removed. The pancreas was divided into three parts, and its tail was used for hematoxylin and eosin coloration. Images were obtained thanks to the Zeiss Axio Scope A.1 microscope (40x/0.75), the Zeiss Axiocam MRc5 Microscope Camera (0.63x) and the software Zen (20 ms of exposure). Scale bar = 50 *μ*m. (l) The percentage of regular and irregular islets was obtained using Visiopharm software. *p* = 0.052 and *p* = 0.078 show the difference between the T1D group and the CTL and T1D GABA groups (ANOVA-Welch). (m) The percentage of infiltrated islets was calculated using the Digital Image Hub software from Leica. Each black point represents an individual. The statistics were performed using a Kruskal-Wallis test. The bars represent the standard error of the mean. For each protocol, the number of mice per group is indicated (*n* = 3 or 6 and *n* = 6). For each mouse, two slides with two tissue sections were analyzed.

**Figure 5:**
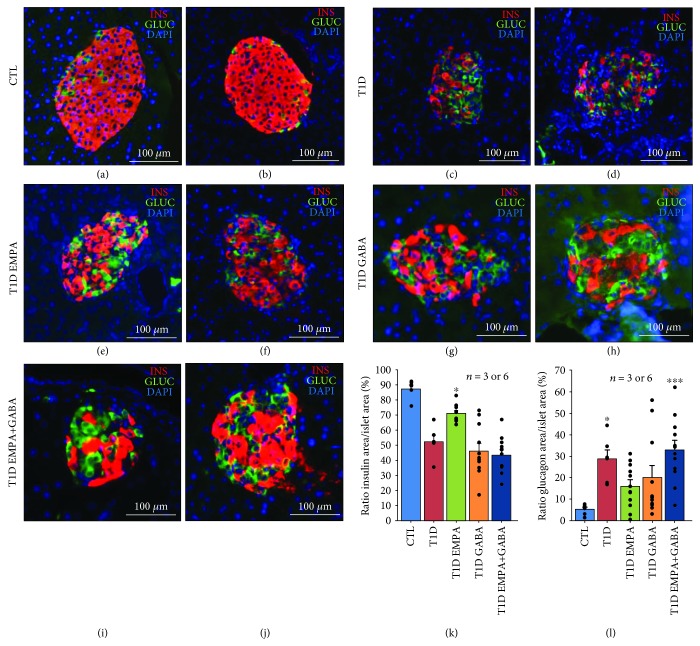
Effect of empagliflozin, GABA, and their combination on the Langerhans islet preservation. At the end of the treatment, the pancreas of control mice (CTL) (a and b); untreated diabetic mice (T1D) (c and d); and mice treated with empagliflozin (T1D EMPA) (e and f), GABA (T1D GABA) (g and h), and both drugs (T1D EMPA+GABA) (i and j) were removed. The pancreas was divided into three parts, and its tail was used for insulin and glucagon staining. The red, green, and blue staining corresponds to insulin, glucagon, and DAPI (nucleus), respectively. Scale bar = 100 m. For protocol #2, the ratio of insulin (k) or glucagon (l) area per islet area was calculated for control mice (CTL, blue bar) and diabetic mice (T1D, red bar) treated with empagliflozin (T1D EMPA, green bar), GABA (T1D GABA, orange bar), or both (T1D EMPA+GABA, navy blue bar), thanks to the 3DHistech Pannoramic P250 Flash III slide scanner and Visiopharm software. (k) Asterisk (^∗^) shows a significant difference (*p* < 0.05; Kruskal-Wallis) between the T1D EMPA group and the T1D GABA and T1D EMPA+GABA groups. (l) Asterisks (^∗^ and ^∗∗∗^) show a significant difference (*p* = 0.014 and *p* = 0.001, respectively; Kruskal-Wallis) between the CTL group and the T1D and T1D EMPA+GABA groups. Each black point represents an individual. The bars represent the standard error of the mean. For each protocol, the number of mice per group is indicated (*n* = 3 or 6). For each mouse, two slides with two tissue sections were analyzed.

Although we observed a deterioration of the islet structure in T1D mice by using H&E staining, mice within the T1D EMPA group had strictly preserved oval-shaped islets such as those in CTL mice (Figure 4(a)–(j)). CTL (82 ± 3%; *p* = 0.052) and T1D GABA_ld_ (77 ± 5%; *p* = 0.076) mice had more round-shaped islets than T1D mice (52 ± 11%; *p* ≤ 0.05), whereas T1D EMPA_ld_ (61 ± 3%; *p* = 0.869) and T1D EMPA+GABA_ld_ (72 ± 7%; *p* = 0.190) mice tended to have improved round-shaped islet proportions compared to T1D mice (Figure 4(l)). Furthermore, most islets in the T1D (21 ± 13%; *p* = 0.143) mice group were infiltrated by immune cells compared to those in the CTL (0 ± 0%) or T1D EMPA (0 ± 0%) mice groups (Figure 4(k) and (m)). The T1D mice group lacked insulin protein expression as seen using immunofluorescence (Figure 5(c) and (d)). Furthermore, islets from mice within the T1D EMPA group showed significantly less *β*-cell loss, as compared to T1D mice (Figure 5). However, diabetic mice from all subgroups contained more glucagon-positive cells than CTL mice (Figure 5). We calculated the ratios between insulin or glucagon areas and islet areas to evaluate *β*- and *α*-cell proportions. The T1D EMPA_ld_ mice contained more insulin-positive cell areas (72 ± 1.62%) (*β*-cell mass) compared to T1D_ld_ (53 ± 4.23%; *p* = 0.502), T1D GABA_ld_ (47 ± 5.20%; *p* = 0.014), and T1D EMPA+GABA_ld_ (44 ± 3.38%; *p* = 0.002) mice (Figure 5(k)). Also, mice from the CTL group (5 ± 0.97%) had lower glucagon areas compared to those from the T1D_ld_ (29 ± 4.315%; *p* = 0.014) and T1D EMPA+GABA_ld_ (33 ± 4.36%; *p* = 0.001) groups (Figure 5(l)). We observed Ki67-positive cells scattered in islets of GABA-treated T1D mice (Figure S2). Interestingly, no Ki67^+^ cells were observed in any other conditions.

**Figure 6:**
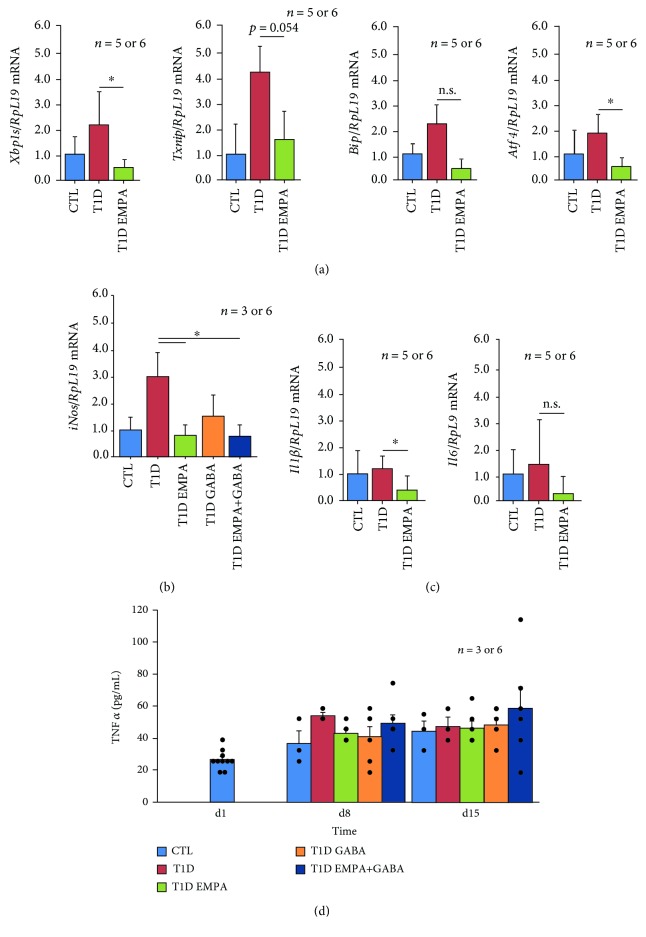
Effect of empagliflozin and GABA on the expression of ER stress, oxidative stress, and inflammation markers. At the end of the treatment, the pancreas of control mice (CTL, blue bar); untreated diabetic mice (T1D, red bar); and mice treated with empagliflozin (T1D EMPA, green bar), GABA (T1D GABA, orange bar), and both (T1D EMPA+GABA, navy blue bar) were taken. The pancreas was divided into three parts, and its body was used for RT-qPCR for the expression of ER stress (a), oxidative stress (b), and inflammation (c) markers. Asterisk (^∗^) shows a significant difference (*p* < 0.05) between T1D mice and T1D EMPA or T1D EMPA+GABA mice. n.s. means nonsignificant. (d) During the studies, blood was collected at different time points in order to analyze TNF*α* secretion, an inflammation marker. There is no experimental difference between the five different samples at day 1; thus, they were displayed as one sample. Each black point represents an individual. The bars represent the standard error of the mean. For each protocol, the number of mice per group is indicated (*n* = 5 or 6 and *n* = 3 to 6). Statistical analyses were performed using a *t*-test for the qPCR results or using a Kruskal-Wallis test for the d15 glucagon results or using an ANOVA-Tukey test for the d22 glucagon results.

To evaluate whether empagliflozin and GABA affected *β*-cell mass through a decrease of endoplasmic reticulum (ER) stress, oxidative stress, and inflammation, we studied these parameters using qPCR. As shown in Figure 6(a), the *Xbp1* spliced form, *Txnip*, *Bip*, and *Atf4* mRNA levels in T1D EMPA mice were decreased compared to those in T1D mice (*p* = 0.041, *p* = 0.054, nonsignificant, and *p* = 0.042, respectively). Expression of these ER stress marker genes was not reduced by treatments GABA (data not shown). A key actor of the oxidative stress is iNOS (inducible nitric oxide synthase) [29, 30]. Its expression was decreased in diabetic mice after empagliflozin and GABA treatment (*p* = 0.025 and *p* = 0.027, respectively) (Figure 6(b)). The expression of two proinflammation cytokines (*Il6* and *Il1β*) in the T1D EMPA group was decreased compared to that in the T1D group (*p* = 0.045 and nonsignificant, respectively) (Figure 6(c)). The expression of both cytokines was not affected by GABA treatment (data not shown).  Figure 6(d) shows that circulating TNF*α* levels were slightly increased between d1 and d8-d15 for all conditions. At d8, T1D EMPA mice tended to have a lower TNF*α* level than untreated T1D mice (43.42 ± 2.20 vs. 54.33 ± 2.15 pg/mL; *p* = 0.306). However, TNF*α* levels were increased in the CTL and T1D EMPA+GABA groups at d22. Taken together, these results indicated that empagliflozin protected *β*-cell mass against glucotoxicity through a decrease of ER stress, oxidative stress, and inflammation gene expression.

## 4. Discussion

Here, we demonstrate that empagliflozin exerts a protective role on *β*-cell mass after T1D onset, and that a 21-day treatment with GABA also improved *β*-cell mass in diabetic mice through *α*-cell proliferation and alleviation of *iNos* expression within islet cells.

Gliflozins are regarded as a promising adjuvant therapy in T1D, since clinical trials (using empa-, sota-, dapa-, or canagliflozin) revealed a positive impact on HbA_1C_ levels, weight management, and daily insulin doses during treatment protocols from 4 to 26 weeks [6–8, 31, 32]. Preclinical data from our study and from the work of Cheng et al. [10] confirmed these clinical findings by showing *β*-cell mass protection against ER stress, oxidative stress, and apoptosis after empagliflozin treatment in T1D-like diabetic mice. It is remarkable that the clinical effects of gliflozins were observed only after short periods of treatment. In our study, we decided to treat T1D mice for a period of 5 days, which was in accordance with previous preclinical studies [9, 10, 33].

The effects of empagliflozin on FFA levels that we observed were in agreement with the study from the group of Jurczak et al. [34] that showed, using hyperglycemic clamps, a twofold increase in insulin levels in *db*/*db*-SGLT2^−/−^ mice compared to control (*db*/*db*-SGLT2^+/+^) mice [34]. In these mice, improvements of *β*-cell function were associated with a 63% increase of *β*-cell volume, compared to *db*/*db*-SGLT2^+/+^ mice. This was due to an increase of islet size—not islet number—and to a decrease of *β*-cell death (*db*/*db*-SGLT2^−/−^ mice showed 64% less TUNEL-positive *β*-cells compared to *db*/*db*-SGLT2^+/+^ mice) [34]. Our results demonstrated that empagliflozin decreased the expression of key ER stress markers (*Xbp1s*, *Bip*, *Atf4*, and *Txnip*) in empagliflozin-treated diabetic mice. This was consistent with the results of Zhou and Wu's study, which demonstrated that empagliflozin improved cardiac function through *Xbp1* and *Atf4* gene expression and through a decrease of BIP and CHOP protein intracellular levels [33].

Moreover, empagliflozin acted on oxidative stress, specifically through a repressive effect on *iNos* expression. In fact, these effects were already observed in obese rats treated with dapagliflozin, which had decreased iNOS protein levels compared to controls [35]. Jaikumkao et al. demonstrated that dapagliflozin improved renal functions in obese prediabetic rats, among other things, by its action on oxidative stress [35]. The reduction of oxidative stress could also be correlated to improved *β*-cell function and glucose homeostasis [36] through a direct effect on IL1*β* signaling as shown *in vitro* on RIN-r cells [37]. This effect of empagliflozin is essential since oxidative stress is key in *β*-cell dysfunction [38, 39] and in diabetes-related vascular complications [40, 41]. In our study, we also observed that empagliflozin acted on inflammation through a decrease of pancreatic *Il1β* and *Il6* expression, which corroborated previous data in diabetic mice (STZ-induced [42, 43] or Akita [44]) that indicated a decrease of *Il1β* or *Il6* mRNA levels after treatment with empagliflozin or dapagliflozin. In our setting, empagliflozin was associated to a trend towards decreased TNF*α* serum levels at d8. These results were consistent with previous data reporting decreased TNF*α* protein levels in STZ-induced diabetes rat or in obese mice or in rats treated with empagliflozin or dapagliflozin [35, 43, 45].

The potential to translate *α*-to-*β* transdifferentiation into clinical protocols for patients with diabetes has become a reality since the recent demonstration of the effects of GABA by Ben-Othman et al. [13]. In our study, only a long-duration treatment of diabetic mice with GABA improved glucose tolerance, islet area/pancreas area ratio, and islet number/pancreas area ratio, with decreased FFA levels. These features are associated with a tendency towards increased C-peptide levels and an increased proliferation of *α* cells and higher *α*-cell density, which confirmed previous works [13]. Effectively, Ben-Othman et al.'s study showed that GABA treatment (250 *μ*g/kg) for a period of 2 to 6 months in healthy mice induced *β*-cell proliferation and Langerhans islet hyperplasia. After STZ-induced *β*-cell apoptosis, GABA treatment allowed *β*-cell mass resurgence [13]. An older study supported Ben-Othman et al.'s work and demonstrated that GABA could reverse STZ-induced diabetes in a mouse model through replication and survival of *β*-cell mass and reduction of inflammation (IL1*β*, TNF*α*, and IFN-*γ*) [46]. Furthermore, GABA regulated cytokine secretion from PBMCs (peripheral blood mononuclear cells) and CD4^+^ T cells [15]. Here, we did not observe the effects of GABA on inflammation, yet we noticed that a long-duration GABA treatment improved oxidative stress within the pancreas, as recently shown by Tang et al. on RINm5F insulinoma cell lines exposed to H_2_O_2_ [47, 48]. Further studies are needed to clarify the role of GABA for *β*-cell mass preservation in patients with T1D.

In our *in vivo* protocol, we decided, in accordance with the ethical committee, to supplement T1D mice (untreated or treated) with 6 U of subcutaneous glargine insulin when blood glucose levels exceeded 400 mg/dL, to ensure the survival and healthy status of the mice. Correlation analyses confirmed that T1D mice injected with exogenous human insulin did not show an improvement in the parameters of *β*-cell survival, confirming independent effects of treatment protocols in these animals. Exogenous insulin supplements are regularly required in diabetic mouse models, in contexts of the evaluation of treatment protocols with progressive efficacy on insulin secretion. For example, in a setting of immature pancreatic precursor cell transplantation into streptozotocin-induced diabetes SCID mice, Rezania et al. relied on exogenous slow-release insulin pellets to ensure graft survival and functionality [49]. In a seminal study evaluating the reprogramming of adult pancreatic exocrine cells into *β*-cells, Zhou et al. induced transdifferentiation using a three-transcription-factor combination after near-to-total *β*-cell depletion, and this protocol required exogenous insulin for maintenance of normoglycemia during the reprogramming process [50]. In our study, we decided to test the effects of empagliflozin in an adjuvant therapy in a preclinical setting mimicking new-onset T1D exempt of severe glucose excursions because of the exogenous insulin treatment. For all these reasons, we decided thus to limit severe hyperglycemia by the adjunction of insulin glargine into the protocol, when required.

Long-term toxic effects of FFAs on *β*-cell function and survival are well known [26, 27] and imply mechanisms of oxidative and endoplasmic reticulum (ER) stress and inflammation [51, 52]. Whether these effects might be involved in the pathophysiology of T1D is currently unclear. Yet, a positive impact on *β*-cell mass and function of fenofibrate, a peroxisome proliferator activator receptor-*α* (PPAR-*α*) agonist, was recently described in a LPL^+/-^ hyperlipidemic mouse model after 8 weeks of treatment [53]. In our study, we also treated diabetic mice with fenofibrate (date not shown). These mice did not show an improvement in glucose homeostasis and tolerance although FFA levels decreased. Still, fenofibrate improved islet area/pancreas area and islet number/pancreas area ratios, and diabetic mice treated with fenofibrate had oval-shaped islets with residual insulin-positive cells. Globally, fenofibrate kept *β*-cell mass functional such as demonstrated by Zheng et al.'s group in a lipoprotein lipase knockout C57BL/6J mice model [53].

## 5. Conclusion

In conclusion, we showed in a preclinical model of T1D that empagliflozin and GABA_ld_ improved glucose homeostasis, islet density and insulin area/islet area ratios, and lipid metabolism through a reduction of ER and oxidative stress within the pancreas. Further investigations are essential to better understand empagliflozin and GABA effects during longer treatment periods and evaluate a potential treatment synergy, since empagliflozin and GABA could be a potential therapeutic treatment to protect *β*-cell mass from destruction after diagnosis of T1D.

## Data Availability

The glycemia monitoring; FFA, glucagon, and TNF dosage; H&E coloration; insulin and glucagon staining; islet area/pancreas area ratio; islet number/pancreas area ratio; infiltration area/islet area ratio; regular islet/irregular islet ratio; insulin area/islet area ratio; glucagon area/islet area ratio; and ER stress, oxidative stress, and inflammation marker gene expression data used to support the findings of this study are included within the article. The insulin dosage, insulin injection evaluation, and Ki-65 staining data used to support the findings of this study are included within the supplementary information file.

## Supplementary Materials

Supplementary Materials Figure S1 shows the insulin level during the IPGTT assay (A). This figure also shows insulin dose injected into diabetic mice through the protocol (B) and glargine insulin (Lantus®, Lilly) which is detected by the ELISA kit (C). Figure S2 shows immunofluorescence with Ki-67 and insulin staining. Table S1 shows primers used for real-time RT-PCR analysis. This table supports data presented in Figure 6. Table S2 shows antibodies used for immunofluorescence staining. This table supports data presented in Figure 5 and Supplementary Figure 2.
