# Supplementary material for: Early Treatment with Empagliflozin and GABA Improves β-Cell Mass and Glucose Tolerance in Streptozotocin-Treated Mice
Source: J Diabetes Res. 2019 Jul 30;2019:2813489. doi: 10.1155/2019/2813489 (PMC6701376; doi:10.1155/2019/2813489)
Supplement: Supplementary Materials — Figure S1 shows the insulin level during the IPGTT assay (A). This figure also shows insulin dose injected into diabetic mice through the protocol (B) and glargine insulin (Lantus®, Lilly) which is detected by the ELISA kit (C). Figure S2 shows immunofluorescence with Ki-67 and insulin staining. Table S1 shows primers used for real-time RT-PCR analysis. This table supports data presented in Figure 6. Table S2 shows antibodies used for immunofluorescence staining. This table supports data presented in Figure 5 and Supplementary Figure 2. [file 2813489.f1.pdf]

Supplemental Information

Early Treatment With Empagliflozin And GABA Improves β-Cell Mass And Glucose Tolerance In Streptozotocin-Treated Mice

Caroline Daems, Sophie Welsch, Hasnae Boughaleb, Juliette Vanderroost, Annie Robert, Etienne Sokal, Philippe A. Lysy

Supplemental Information inventory:

Figure S1 shows insulin level during the IPGTT assay (A). This figure also shows insulin dose injected to the diabetic mice through the protocol (B and C).

Figure S2 shows immunofluorescence with a Ki-67 and insulin staining.

Table S1 shows primers used for real-time RT-PCR analysis. This table supports data presented in main Figure 7.

Table S2 shows antibodies used for immunofluorescence staining. This table supports data presented in main Figure 6 and supplemental Figure 2.

Supplemental Materials & Methods

Intraperitoneal glucose tolerance test (IPGTT)

IPGTTs were performed at the end of the treatment protocols (day 12 or 28). Blood samples were collected at 0, 30 and 60 min during IPGTT and were used to measured insulin levels using a commercial ELISA kit (Mercodia), following the manufacturer's instructions. Results were acquired using a spectrophotometer (VictorTM X4, 2030 Multilabel Reader, Perkin Elmer) at 450 nm.

Immunofluorescence

Pancreas tissues were fixed overnight in 4% paraformaldehyde (PFA) and embedded in paraffin. Then, the samples were sectioned into 5 μm thick slices, blocked in 10% bovine serum albumin (Sigma-Aldrich), 3% no fat milk and 0.3% Triton X-100 PBS. For the detection of Ki67, tissues were incubated overnight at 4°C with antibody (listed in **Table S2**) diluted in PBS/0.3% Triton X-100/10% BSA/3% milk. Then tissues were incubated the next day for 2 h at RT in the dark with respective secondary antibody (listed in **Table S2**) diluted in PBS/0.3% Triton X-100/10% BSA. After washing, tissues were incubated for 5 min with DAPI 397-412 nm diluted to 1:10,000 in distilled water. Then, sections were mounted with Faramount mounting medium (Dako, # S3025) and observed under a fluorescence microscope. The digital images were acquired by a digital imaging platform (3DHistech Pannoramic P250 Flash III, nicknamed Oyster).

35 **Figure S1.**

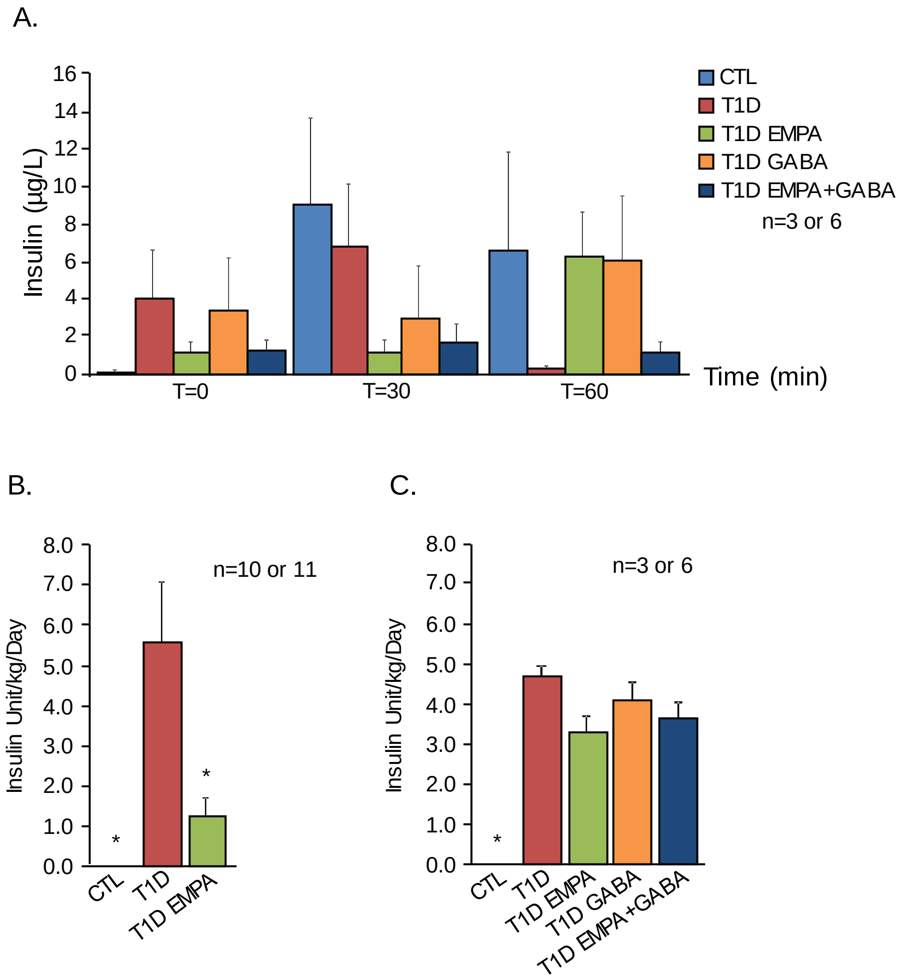

A.
Insulin (µg/L)
16
14
12
10
8
6
4
2
0
T=0
T=30
T=60
Time (min)
CTL
T1D
T1D EMPA
T1D GABA
T1D EMPA+GABA
n=3 or 6
B.
Insulin Unit/kg/Day
8.0
7.0
6.0
5.0
4.0
3.0
2.0
1.0
0.0
n=10 or 11
*
*
CTL
T1D
T1D EMPA
C.
Insulin Unit/kg/Day
8.0
7.0
6.0
5.0
4.0
3.0
2.0
1.0
0.0
n=3 or 6
*
CTL
T1D
T1D EMPA
T1D GABA
T1D EMPA+GABA

Figure S1. Daily dose of insulin received by the mice through the study. (A) At 0, 30 and 60 min post glucose injection, blood of control mice (CTL, blue bar), untreated diabetic mice (T1D, red bar), diabetic mice treated with empagliflozin (T1D EMPA, green bar), GABA (T1D GABA, orange bar) or empagliflozin and GABA (T1D EMPA+GABA, navy blue bar) was collected. Insulin level was evaluated by ELISA kit. The bars represent the standard error of the mean. Number of mice per group is indicated (n=3 or 6). The statistics were performed using a ANOVA-Tukey test for the times 0 and 30 min and using a Kruskal-Wallis for the time 60 min. (B and C) Daily, human Lantus insulin was injected in control mice (CTL, blue bar), untreated diabetic mice (T1D, red bar), diabetic mice treated with empagliflozin (T1D EMPA, green bar), GABA (T1D GABA, orange bar) or empagliflozin and GABA (T1D EMPA+GABA, navy blue bar). Diabetic mice received 6 unit of Human Lantus insulin by subcutaneous injection when their glycemia was higher than 400 mg/dL. B and C correspond to the protocol #1 and #2, respectively. (B) The asterisk (*) shows a significant difference ($p=0.004$; ANOVA+Tukey) between T1D group and CTL and T1D EMPA groups. (C) The asterisk (*) shows a significant difference ($p<0.001$; ANOVA-Tukey) between CTL group and T1D, T1D EMPA, T1D GABA and T1D EMPA+GABA groups. The bars represent the standard error of the mean. For each study, number of mice per group is indicated (n=10 or 11, n=3 or 6).

Figure S2.

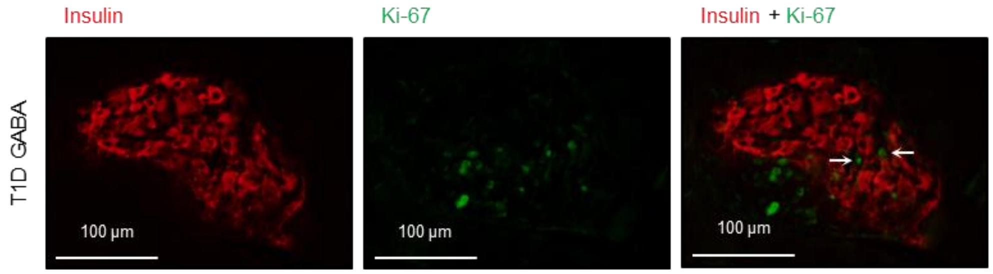

Figure S2. Effect of GABA on α cells proliferation. The pancreas of T1D GABA mice was divided into three parts and its tail was used for insulin and Ki-67 staining. The red and green staining corresponds to the insulin and Ki-67, respectively. Arrows indicated Ki-67 positive nucleus. Images were obtained thank the microscope Scope A.1 Zeiss (40x/0.75), the camera Axiocam MRc5 Zeiss (0.63x) and the software Zen (Insulin: 250 ms and Ki-67: 300 ms). Scale bar=100 µm.

Table S1. SYBER Green Primers

Function	Gene symbol		Sequence	Ref
Oxidative stress	*iNOS*	Forward:	5’-AATCCCTGGACAAGCTGCAT-3’	
		Reverse:	5’-TCTGGTCAAACTCTTGGGGTT-3’	
Stress ER	*sXbp1*	Forward:	5’-CTGAGTCCGCAGCAGGTGCAG-3’	[1]
		Reverse:	5’-GTCCATGGGAAGATGTTCTGG-3’	
	Atf4	Forward:	5’-GGGTTCTGTCTTCCACTCCA-3’	[1]
		Reverse:	5’-AAGCAGCAGAGTCAGGCTT(TC)-3’	
	Bip	Forward:	5’-TTCAGCCAATTATCAGCAAACTCT-3’	[1]
		Reverse:	5’-TTTTCTGATGTATCCTCTTCACCAGT-3’	
	Txnip	Forward:	5’-AAGTTACCCGAGTCAAAGCCGT-3’	
		Reverse:	5’-TTCCAGGCCTCATGATCACCAT-3’	
Inflammation	*IL1β*	Forward:	5’-ATCTGGGATCCTCTCCAGCCAA-3’	
		Reverse:	5’-TCATCAGGACAGCCCAGGT-3’	
	IL6	Forward:	5’-AACCGCTATGAAGTTCCTCTCT-3’	
		Reverse:	5’-AGGTCTGTTGGGAGTGGTA-3’	
Internal control	*RpL19*	Forward:	5’-CTGAAGGTCAAAGGGAATGTG-3’	[2]
		Reverse:	5’-GGACAGAGTCTTGATGATCTC-3’	

Table S2. Antibodies

Antibody	Firm	Reference	Dilution
Insuline	Cell Signaling	3014S	1:500
Insuline	Dako	A0564	1:500
Glucagon	Santa Cruz	sc-71152	1:400
Ki67	ThermosFisher	MA5-14520	1:250
Anti rabbit	Life technologies	A11012	1:1,000
Anti mouse	Life technologies	A11001	1:1,000
Anti guinea pig	Life technologies	A21450	1:1,000

References

1. Oslowski, C.M. and F. Urano, *Measuring ER stress and the unfolded protein response using mammalian tissue culture system.* Methods Enzymol, 2011. **490**: p. 71-92.
2. Guigon, C.J., et al., *Follicular cells acquire sertoli cell characteristics after oocyte loss.* Endocrinology, 2005. **146**(7): p. 2992-3004.
